# Bis(2,3,5-triphenyl­tetra­zolium) tetra­thio­cyanato­cobaltate(II)

**DOI:** 10.1107/S1600536809041464

**Published:** 2009-10-23

**Authors:** Kouichi Nakashima, Naoyuki Kawame, Yoshiko Kawamura, Osamu Tamada, Jun Yamauchi

**Affiliations:** a Graduate School of Human and Environmental Studies, Kyoto University, Yoshidanihonmatsu-cho, Sakyo-ku, Kyoto 606-8501, Japan; b Department of Bio-Environmental Sciences, Kyoto Gakuen University, Sogabecho-Kameoka, Kyoto 621-8555, Japan; c Graduate School of Science, Kyoto University, Oiwake-cho, Kitashirakawa, Sakyo-ku, Kyoto 606-8502, Japan

## Abstract

The title compound, (C_19_H_15_N_4_)_2_[Co(NCS)_4_], has two crystallographycally different molecules of bis­(2,3,5-triphenyl­tetra­zolium) tetra­thio­cyanatecobaltate in the asymmetric unit. There are only minor geometric differences between them. Each cobalt(II) ion is coordinated by the N atoms of four NCS anions, showing the magnitude of the magnetic moment expected from the NCS^−^ crystal field strength.

## Related literature

For the use of tetra­zolium complexes in studying enzymatic redox reactions, see: Saide & Gilliland (2005 ▶). For studies of tetra­zolium complexes and cobaltate compounds, see: Matulis *et al.* (2003 ▶); Kawamura *et al.* (1997 ▶); Rizzi *et al.* (2003 ▶); Marzotto *et al.* (1999 ▶); Fukui *et al.* (1992 ▶); Kubo *et al.* (1979 ▶). For the structures of tetra­zolium complexes, see: Matulis *et al.* (2003 ▶); Kawamura *et al.* (1997 ▶). For the structure of tetra­ethyl­ammonium tetrachloridonickelate(II), see: Stucky *et al.* (1967 ▶). For the magnetic moment as a measure of the crystal field strength, see: Van Vleck (1932 ▶); Ballhausen (1962 ▶). For a bis­(formaza­nato) cobalt(II) complex in which the cobalt(II) ion is in a low spin state, see: Kawamura *et al.* (1990 ▶). 1,3,5-Triphenyl­formazan, used in the preparation of the title compound, is well known to be oxidized to the corresponding tetra­zolium cation by utilizing some oxidation reagent or air oxidation, see: Nineham (1955 ▶).
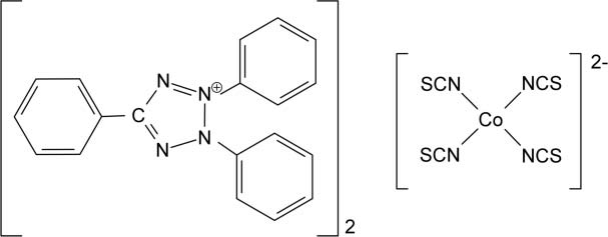


## Experimental

### 

#### Crystal data

(C_19_H_15_N_4_)_2_[Co(NCS)_4_]
*M* *_r_* = 889.99
Monoclinic, 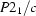

*a* = 9.5667 (2) Å
*b* = 49.7156 (11) Å
*c* = 18.9036 (7) Å
β = 102.810 (3)°
*V* = 8767.0 (4) Å^3^
*Z* = 8
Mo *K*α radiation
μ = 0.63 mm^−1^
*T* = 298 K
0.26 × 0.22 × 0.10 mm

#### Data collection

Nonus KappaCCD diffractometer
Absorption correction: Gaussian (**WinGX** routine *Gaussian*; Farrugia, 1999 ▶; Coppens *et al.*, 1965 ▶) *T*_min_ = 0.854, *T*_max_ = 0.938
63412 measured reflections
15338 independent reflections
8769 reflections with *I* > 2σ(*I*)
*R*_int_ = 0.043

#### Refinement

*R*[*F* ^2^ > 2σ(*F* ^2^)] = 0.049
*wR*(*F* ^2^) = 0.121
*S* = 1.03
15338 reflections
1063 parameters
H-atom parameters constrained
Δρ_max_ = 0.34 e Å^−3^
Δρ_min_ = −0.33 e Å^−3^

### 

Data collection: *COLLECT* (Nonius, 1998 ▶); cell refinement: *DENZO-SMN* (Otwinowski & Minor, 1997 ▶); data reduction: *DENZO-SMN* and *SORTAV* (Blessing, 1987 ▶; Blessing & Langs, 1987 ▶); program(s) used to solve structure: *SIR97* (Altomare *et al.*, 1999 ▶); program(s) used to refine structure: *SHELXL97* (Sheldrick, 2008 ▶); molecular graphics: *ORTEP-3 for Windows* (Farrugia, 1997 ▶); software used to prepare material for publication: *WinGX* (Farrugia, 1999 ▶).

## Supplementary Material

Crystal structure: contains datablocks global, I. DOI: 10.1107/S1600536809041464/bt5038sup1.cif

Structure factors: contains datablocks I. DOI: 10.1107/S1600536809041464/bt5038Isup2.hkl

Additional supplementary materials:  crystallographic information; 3D view; checkCIF report

## Figures and Tables

**Fig. 1.**
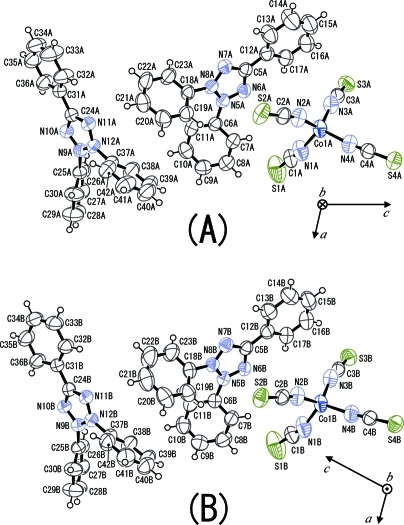
The geometry and the numbering scheme of the complex units A(A) and B(B). Displacement ellipsoids are shown at the 30% probability level. H atoms are shown as small spheres of arbitrary radii.

**Fig. 2.**
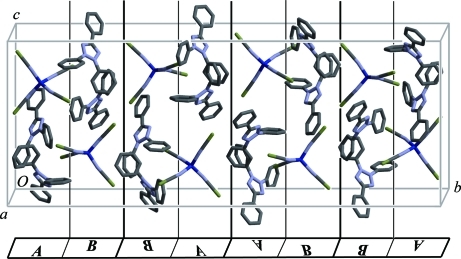
The crystal packing in the unit cell of (C19N4H15)2Co(NCS)4. The arrangement of the two distinct (C19N4H15)2Co(NCS)4 complexes are illustrated at the bottom as A and B columns in the monoclinic unit cell. H atoms are omitted for clarity.

## supplementary crystallographic information

### Comment

Tetrazolium complexes, such as triphenyltetrazolium chloride (TTC), are highly sensitive color indicators of enzymatic redox reactions, and they are used in studies of such reactions (Saide & Gilliland, 2005). Several studies have been conducted on tetrazolium complexes and cobaltate compounds (Matulis *et al.*, 2003; Kawamura *et al.*, 1997; Rizzi *et al.*, 2003; Marzotto *et al.*, 1999; Fukui *et al.*, 1992; Kubo *et al.*, 1979), and a few structures of tetrazolium complexes have been determined (Matulis *et al.*, 2003; Kawamura *et al.*, 1997). Kawamura *et al.* (1997) studied the crystal structure of a complex composed of a 2,3,5-triphenyltetrazolium cation and a dichloro(1,3,5-triphenylformazanato) cobaltate (II) anion (hereafter designated as complex Type I), with the magnetic properties of Co(II) by means of a superconducting quantum-interference device (SQUID) and electron spin resonance (ESR) spectroscopy. In this communication, we report the crystal structure of bis(2,3,5-triphenyltetrazolium) tetrathiocyanocobaltate ((C_19_N_4_H_15_)_2_Co(NCS)_4_), determined from single-crystal X-ray diffraction data, and compare its structure and physical properties to those of the Type I complex.

Since the coordination of Co(II) is an important factor for many physical properties, one objective of the current study was to clarify the ligands around the Co(II) ion, Co(NCS)_4_^2-^ or Co(SCN)_4_^2-^; the coordination with four N atoms from NCS anions was confirmed to be Co(NCS)_4_^2-^. Furthermore, the crystallographic result revealed an asymmetric unit composed of a pair of units of (C_19_N_4_H_15_)_2_Co(NCS)_4_ and a successive array of four asymmetric units in the b direction with alternating orientation. Both units in an asymmetric moiety are structurally different although the difference is subtle, and, therefore crystallographically distinct. Hereafter, they are referred to as A and B. Within them, an anion, Co(NCS)_4_^-^, and two cations, both C_19_N_4_H_15_^+^, would interact as a result of interionic force. The geometry and atomic numbering schemes for A and B complexes are shown in Fig. 1.

The current one exhibited a typical cobalt-blue color because the tetrazolium cation does not have any absorption in the visible range. On the other hand, the ligand formazan molecule has strong absorption of about 580 nm and the color of the Type I complex was almost black. As a result, the absorptions around the Co(II) ion could not be assigned and it was impossible to compare the crystal field strength of the two based upon the absorption. Referring to the bond distances, it could be described that the crystal field of the current one might be stronger than that of the Type I complex, since the shorter distances provide a smaller Co(II) tetrahedral volume than that of the Type I complex.

The magnitude of the magnetic moment also measures the crystal field strength because the crystal field strength is incorporated in magnetic moment; it is generally correct to mention that the larger is the crystal field, the smaller is the magnetic moment (Van Vleck, 1932; Ballhausen, 1962). The magnetic moments of the Type I complex and the current one at room temperature were 4.0 µ_B_ and 4.5 µ_B_, which correspond to the larger and smaller crystal fields, respectively. Therefore, the order is opposite to what is predicted from the structural analysis. In fact, the magnetic moments of the complexes of CoCl_4_^2-^, CoBr_4_^2-^, and CoI_4_^2-^, with the triphenyltetrazolium cation were 4.7, 5.0, and 5.2 µ_B_, respectively, and this order corresponds with the inverse of the crystal field strength. And the current complex appropriately followed the order.

One of the authors observed a low spin state of the cobalt(II) ion in the bis(formazanato) cobalt(II) complex on ESR and magnetic susceptibility measurements, and the coordination was supposed to be from four N atoms of two formazan molecules (Kawamura *et al.*, 1990). The fact suggests the larger crystal field and supports the magnitude of the magnetic moment of the Type I complex. Therefore, formazan molecule might provide somewhat stronger coordination than that expected from the structural analysis and lead to the smaller magnetic moment in the Type I complex. It would be correct to state that the crystal field strengths of the present two complexes would follow the order.

The coordination of Co(II) is an important factor in the magnetic, optical absorption (colour) and ESR properties. The Co(II) ion is four-coordinated in the both structures. In the Type I complex, two of the coordinating ligands are N (with an average Co—N distance of 1.959 Å) and two are Cl (with an average Co—Cl distance of 2.248 Å). The average bond distance to Co(II) in the Type I complex is thus 2.104 Å. The two N atoms are members of formazan, that comprise a large complex merged by the triphenyltetrazolium and Co(NCl)^2-^. The absorption bands of Co(II) ion are not separated due to the strong absorption of formazan. Therefore,the colour of Type I is (almost) black due to the absorption overlapping of Co(II) ion and formazan molecule. In (C_19_N_4_H_15_)_2_Co(NCS)_4_, the two distinct Co(NCS)_4_^2-^ anionic complexes have average Co—N distances of 1.948 Å and 1.947 Å, thus yielding a much smaller Co(II) tetrahedral volume and stronger crystal field compared to the Type I complex. Individual Co(II) ions are separated by more than 11 Å from each other in the structure, thus each Co(NCS)_4_^2-^ complex behaves as a magnetically isolated entity. The crystal exhibits a typical cobalt-blue colour because of the absence of formasan molecule. However, it is impossible to have some comparison about the crystal field difference of the two based upon the absorptions because of the lack of the clear absorption due the cobalt ion in the Type I complex.

Furthermore, the 1,3,5-triphenyltetrazolium ion is also bulkier as the counter ion and very flexible due to the three phenyl groups.

### Experimental

The reaction mixture of 430 mg of Co(NO_3_)_2_.2H_2_O, 280 mg of KNCS, and 500 mg of 1,3,5-triphenylformazan in 40 ml ethanol were kept standing in room temperature. 1,3,5-triphenylformazan is well known to be oxidized to the corresponding tetrazolium cation by utilizing some oxidation reagent or air oxidation (Nineham, 1955). 1,3,5-triphenyfomazen was likely to be oxidized probably by air to 2,3,5-tetrazolium in the solution, as the result, to form the complex together with tetrathiocyano cobaltate(II) anion. The complex with deep blue color was crystallized in one week. The crystals were filtrated and washed with ethanol. The result of C, H, and N elemental analyses of the complex was in good accordance with the calculated values in bis(2,3,5-triphenyl tetrazolium) tetrathiocyano cobaltate(II), respectively. The crystals were quite stable in air. The results of elemental analyses are followed; Exp. C; 56.12, H; 3.29, N; 18.89%, Calcd; C;56.67, H; 3.37, N;18.89%

### Refinement

All aromatic H atoms were placed in idealized positions and refined as a riding model, with C—H = 0.93 Å and *U*_iso_(H) = 1.2*U*_eq_(C).

### Crystal data

**Table:**

(C_19_H_15_N_4_)_2_[Co(NCS)_4_]	*F*(000) = 3656
*M**_r_* = 889.99	*D*_x_ = 1.349 Mg m^−^^3^
Monoclinic, *P*2_1_/*c*	Mo *K*α radiation, λ = 0.71073 Å
Hall symbol: -P 2ybc	Cell parameters from 14264 reflections
*a* = 9.5667 (2) Å	θ = 1.4–25.0°
*b* = 49.7156 (11) Å	µ = 0.63 mm^−^^1^
*c* = 18.9036 (7) Å	*T* = 298 K
β = 102.810 (3)°	Plate, blue
*V* = 8767.0 (4) Å^3^	0.26 × 0.22 × 0.10 mm
*Z* = 8	

### Data collection

**Table:**

Nonus KappaCCD diffractometer	8769 reflections with *I* > 2σ(*I*)
φ scans, and ω scans with κ offsets	*R*_int_ = 0.043
Absorption correction: gaussian (*WinGX* routine Gaussian; Farrugia, 1999; Coppens *et al.*, 1965)	θ_max_ = 25.0°, θ_min_ = 1.4°
*T*_min_ = 0.854, *T*_max_ = 0.938	*h* = −11→11
63412 measured reflections	*k* = −58→59
15338 independent reflections	*l* = −21→22

### Refinement

**Table:**

Refinement on *F*^2^	0 restraints
Least-squares matrix: full	H-atom parameters constrained
*R*[*F*^2^ > 2σ(*F*^2^)] = 0.049	*w* = 1/[σ^2^(*F*_o_^2^) + (0.0389*P*)^2^ + 4.6897*P*] where *P* = (*F*_o_^2^ + 2*F*_c_^2^)/3
*wR*(*F*^2^) = 0.121	(Δ/σ)_max_ = 0.001
*S* = 1.03	Δρ_max_ = 0.34 e Å^−^^3^
15338 reflections	Δρ_min_ = −0.33 e Å^−^^3^
1063 parameters	

### Special details

**Table:**

Geometry. All e.s.d.'s (except the e.s.d. in the dihedral angle between two l.s. planes) are estimated using the full covariance matrix. The cell e.s.d.'s are taken into account individually in the estimation of e.s.d.'s in distances, angles and torsion angles; correlations between e.s.d.'s in cell parameters are only used when they are defined by crystal symmetry. An approximate (isotropic) treatment of cell e.s.d.'s is used for estimating e.s.d.'s involving l.s. planes.

### Fractional atomic coordinates and isotropic or equivalent isotropic displacement parameters (Å^2^)

**Table:**

	*x*	*y*	*z*	*U*_iso_*/*U*_eq_	
Co1A	0.94227 (6)	0.07649 (1)	0.74245 (3)	0.07538 (16)	
N1A	1.0728 (4)	0.10017 (8)	0.70699 (19)	0.1034 (12)	
N2A	0.8771 (4)	0.05015 (7)	0.66646 (17)	0.0869 (10)	
N3A	0.7998 (4)	0.10109 (7)	0.76653 (17)	0.0867 (10)	
N4A	1.0305 (4)	0.05694 (7)	0.83052 (18)	0.0866 (10)	
N5A	0.7775 (3)	0.07169 (5)	0.44237 (14)	0.0607 (7)	
N6A	0.7174 (3)	0.07705 (6)	0.49722 (14)	0.0641 (7)	
N7A	0.5852 (3)	0.04677 (6)	0.42437 (14)	0.0664 (7)	
N8A	0.6983 (3)	0.05331 (6)	0.39903 (14)	0.0617 (7)	
N9A	1.0664 (3)	0.07658 (6)	0.06199 (15)	0.0618 (7)	
N10A	0.9543 (3)	0.08134 (5)	0.00899 (14)	0.0618 (7)	
N11A	0.9103 (3)	0.04605 (6)	0.07406 (14)	0.0642 (7)	
N12A	1.0394 (3)	0.05548 (6)	0.10107 (14)	0.0622 (7)	
S1A	1.1939 (2)	0.14454 (3)	0.66008 (9)	0.1625 (7)	
S2A	0.85502 (14)	0.01430 (2)	0.55196 (6)	0.0980 (4)	
S3A	0.66767 (12)	0.14517 (2)	0.81374 (6)	0.0892 (3)	
S4A	1.12201 (12)	0.02510 (2)	0.95227 (5)	0.0796 (3)	
C1A	1.1245 (5)	0.11889 (9)	0.6873 (2)	0.0859 (12)	
C2A	0.8677 (4)	0.03516 (8)	0.6186 (2)	0.0741 (10)	
C3A	0.7437 (4)	0.11957 (8)	0.78632 (19)	0.0719 (10)	
C4A	1.0694 (4)	0.04375 (7)	0.8822 (2)	0.0673 (9)	
C5A	0.6001 (4)	0.06141 (7)	0.48595 (17)	0.0613 (9)	
C6A	0.9131 (4)	0.08306 (7)	0.43634 (17)	0.0610 (9)	
C7A	1.0147 (4)	0.08572 (8)	0.49989 (19)	0.0796 (11)	
H7A	0.9961	0.0801	0.5439	0.095*	
C8A	1.1448 (5)	0.09691 (9)	0.4966 (2)	0.0931 (13)	
H8A	1.2141	0.0994	0.5391	0.112*	
C9A	1.1733 (4)	0.10437 (8)	0.4314 (2)	0.0839 (11)	
H9A	1.2628	0.1113	0.4296	0.101*	
C10A	1.0706 (5)	0.10161 (8)	0.3693 (2)	0.0815 (11)	
H10A	1.0903	0.1068	0.3253	0.098*	
C11A	0.9368 (4)	0.09110 (7)	0.37079 (18)	0.0732 (10)	
H11A	0.8657	0.0896	0.3286	0.088*	
C12A	0.5064 (4)	0.05921 (8)	0.53656 (18)	0.0690 (9)	
C13A	0.4026 (5)	0.03948 (9)	0.5285 (2)	0.0947 (13)	
H13A	0.3887	0.028	0.4888	0.114*	
C14A	0.3194 (6)	0.03679 (10)	0.5791 (3)	0.1175 (16)	
H14A	0.2491	0.0235	0.5733	0.141*	
C15A	0.3400 (6)	0.05363 (11)	0.6381 (3)	0.1107 (15)	
H15A	0.2848	0.0516	0.6725	0.133*	
C16A	0.4417 (5)	0.07340 (11)	0.6463 (2)	0.1043 (15)	
H16A	0.4541	0.085	0.6858	0.125*	
C17A	0.5258 (4)	0.07622 (9)	0.5964 (2)	0.0866 (12)	
H17A	0.5958	0.0895	0.6026	0.104*	
C18A	0.7328 (4)	0.04081 (7)	0.33545 (17)	0.0611 (9)	
C19A	0.8543 (4)	0.02553 (7)	0.34416 (19)	0.0728 (10)	
H19A	0.9156	0.0236	0.3895	0.087*	
C20A	0.8829 (4)	0.01316 (8)	0.2838 (2)	0.0820 (11)	
H20A	0.965	0.0027	0.2882	0.098*	
C21A	0.7919 (5)	0.01608 (8)	0.2174 (2)	0.0824 (11)	
H21A	0.812	0.0075	0.177	0.099*	
C22A	0.6722 (5)	0.03150 (9)	0.2104 (2)	0.0957 (13)	
H22A	0.6112	0.0335	0.165	0.115*	
C23A	0.6399 (4)	0.04424 (8)	0.26987 (19)	0.0861 (12)	
H23A	0.558	0.0548	0.2654	0.103*	
C24A	0.8593 (4)	0.06210 (7)	0.01706 (17)	0.0584 (8)	
C25A	1.1996 (4)	0.09142 (8)	0.07227 (17)	0.0641 (9)	
C26A	1.1920 (4)	0.11882 (8)	0.07591 (18)	0.0715 (10)	
H26A	1.1053	0.1275	0.0742	0.086*	
C27A	1.3182 (5)	0.13309 (8)	0.0822 (2)	0.0842 (11)	
H27A	1.3172	0.1518	0.0851	0.101*	
C28A	1.4449 (5)	0.11987 (10)	0.0841 (2)	0.0972 (13)	
H28A	1.5291	0.1297	0.0883	0.117*	
C29A	1.4487 (5)	0.09242 (10)	0.0798 (3)	0.1035 (14)	
H29A	1.5354	0.0837	0.0814	0.124*	
C30A	1.3239 (4)	0.07752 (8)	0.0732 (2)	0.0878 (12)	
H30A	1.3244	0.0589	0.0695	0.105*	
C31A	0.7202 (3)	0.05817 (7)	−0.03147 (17)	0.0602 (8)	
C32A	0.6403 (4)	0.03584 (8)	−0.0238 (2)	0.0900 (12)	
H32A	0.6745	0.0235	0.013	0.108*	
C33A	0.5097 (5)	0.03171 (10)	−0.0704 (3)	0.1140 (16)	
H33A	0.4557	0.0166	−0.0649	0.137*	
C34A	0.4588 (4)	0.04958 (10)	−0.1247 (2)	0.0984 (14)	
H34A	0.3711	0.0466	−0.1565	0.118*	
C35A	0.5366 (5)	0.07175 (10)	−0.1319 (2)	0.0952 (13)	
H35A	0.5016	0.084	−0.1688	0.114*	
C36A	0.6671 (4)	0.07632 (8)	−0.0853 (2)	0.0854 (12)	
H36A	0.7191	0.0917	−0.0904	0.102*	
C37A	1.1342 (4)	0.04383 (8)	0.16388 (18)	0.0668 (9)	
C38A	1.1814 (4)	0.05895 (9)	0.2245 (2)	0.0909 (12)	
H38A	1.1594	0.0772	0.2251	0.109*	
C39A	1.2629 (5)	0.04651 (12)	0.2850 (2)	0.1101 (16)	
H39A	1.2955	0.0564	0.3272	0.132*	
C40A	1.2959 (5)	0.01991 (12)	0.2835 (3)	0.1056 (15)	
H40A	1.3509	0.0118	0.3247	0.127*	
C41A	1.2488 (5)	0.00504 (9)	0.2219 (3)	0.0997 (14)	
H41A	1.2725	−0.0131	0.2212	0.12*	
C42A	1.1657 (4)	0.01702 (9)	0.1607 (2)	0.0838 (11)	
H42A	1.1323	0.0071	0.1185	0.101*	
Co1B	0.19828 (6)	0.17299 (1)	0.23657 (2)	0.07351 (16)	
N1B	0.3640 (4)	0.14947 (8)	0.26138 (19)	0.1087 (12)	
N2B	0.2309 (4)	0.19870 (7)	0.31647 (17)	0.0858 (10)	
N3B	0.0327 (4)	0.14898 (7)	0.22109 (17)	0.0869 (10)	
N4B	0.1743 (3)	0.19403 (7)	0.14799 (17)	0.0831 (9)	
N5B	0.3353 (3)	0.17748 (5)	0.53597 (14)	0.0593 (7)	
N6B	0.2251 (3)	0.17185 (5)	0.48320 (14)	0.0620 (7)	
N7B	0.1622 (3)	0.20269 (5)	0.55616 (14)	0.0633 (7)	
N8B	0.2977 (3)	0.19635 (5)	0.57947 (14)	0.0607 (7)	
N9B	0.9743 (3)	0.17873 (6)	0.91658 (14)	0.0619 (7)	
N10B	0.9168 (3)	0.17294 (5)	0.97168 (14)	0.0610 (7)	
N11B	0.8028 (3)	0.20800 (6)	0.90980 (14)	0.0634 (7)	
N12B	0.9059 (3)	0.19945 (6)	0.87930 (14)	0.0619 (7)	
S1B	0.5377 (2)	0.10818 (3)	0.31845 (10)	0.1738 (8)	
S2B	0.34229 (13)	0.23541 (2)	0.42447 (5)	0.0870 (3)	
S3B	−0.15156 (12)	0.10669 (2)	0.17005 (6)	0.0927 (3)	
S4B	0.11865 (11)	0.22971 (2)	0.03283 (5)	0.0762 (3)	
C1B	0.4375 (4)	0.13202 (9)	0.2852 (2)	0.0824 (11)	
C2B	0.2796 (4)	0.21418 (8)	0.36141 (19)	0.0684 (9)	
C3B	−0.0449 (4)	0.13123 (8)	0.19918 (18)	0.0698 (10)	
C4B	0.1496 (4)	0.20888 (7)	0.09917 (19)	0.0644 (9)	
C5B	0.1193 (4)	0.18782 (7)	0.49594 (17)	0.0594 (8)	
C6B	0.4748 (4)	0.16533 (6)	0.54337 (18)	0.0599 (8)	
C7B	0.5205 (4)	0.16040 (7)	0.48042 (19)	0.0736 (10)	
H7B	0.4632	0.1648	0.4354	0.088*	
C8B	0.6532 (5)	0.14877 (8)	0.4859 (2)	0.0868 (12)	
H8B	0.6852	0.1449	0.4441	0.104*	
C9B	0.7384 (4)	0.14283 (8)	0.5523 (2)	0.0843 (11)	
H9B	0.8292	0.1356	0.5554	0.101*	
C10B	0.6899 (5)	0.14756 (8)	0.6142 (2)	0.0842 (11)	
H10B	0.7479	0.1433	0.6592	0.101*	
C11B	0.5555 (4)	0.15854 (7)	0.61034 (19)	0.0755 (10)	
H11B	0.5209	0.1612	0.652	0.091*	
C12B	−0.0230 (4)	0.18965 (7)	0.44848 (18)	0.0665 (9)	
C13B	−0.1201 (4)	0.20869 (8)	0.4618 (2)	0.0794 (11)	
H13B	−0.0954	0.2199	0.502	0.095*	
C14B	−0.2533 (5)	0.21099 (9)	0.4156 (3)	0.0942 (13)	
H14B	−0.3184	0.2237	0.4247	0.113*	
C15B	−0.2892 (5)	0.19444 (11)	0.3562 (3)	0.0989 (14)	
H15B	−0.3787	0.1961	0.3249	0.119*	
C16B	−0.1938 (5)	0.17546 (11)	0.3427 (2)	0.1022 (14)	
H16B	−0.2192	0.1642	0.3025	0.123*	
C17B	−0.0612 (4)	0.17310 (8)	0.3883 (2)	0.0844 (11)	
H17B	0.0033	0.1603	0.3789	0.101*	
C18B	0.3902 (4)	0.20985 (7)	0.63978 (18)	0.0647 (9)	
C19B	0.5086 (4)	0.22298 (8)	0.6285 (2)	0.0830 (11)	
H19B	0.5332	0.2226	0.5836	0.1*	
C20B	0.5915 (5)	0.23697 (9)	0.6872 (3)	0.1005 (14)	
H20B	0.6732	0.2462	0.682	0.121*	
C21B	0.5516 (6)	0.23708 (9)	0.7526 (3)	0.1071 (16)	
H21B	0.6076	0.2462	0.7917	0.129*	
C22B	0.4327 (6)	0.22416 (11)	0.7611 (2)	0.1180 (17)	
H22B	0.4069	0.2248	0.8057	0.142*	
C23B	0.3488 (5)	0.21001 (9)	0.7050 (2)	0.0963 (13)	
H23B	0.2672	0.2009	0.7109	0.116*	
C24B	0.8121 (4)	0.19133 (7)	0.96704 (17)	0.0596 (8)	
C25B	1.0967 (4)	0.16456 (8)	0.90157 (17)	0.0640 (9)	
C26B	1.0888 (4)	0.13711 (8)	0.89661 (18)	0.0727 (10)	
H26B	1.0064	0.128	0.9012	0.087*	
C27B	1.2070 (5)	0.12342 (9)	0.8846 (2)	0.0856 (11)	
H27B	1.2053	0.1048	0.8814	0.103*	
C28B	1.3261 (5)	0.13732 (10)	0.8776 (2)	0.0997 (14)	
H28B	1.4046	0.128	0.8685	0.12*	
C29B	1.3320 (5)	0.16471 (10)	0.8836 (3)	0.1021 (14)	
H29B	1.4147	0.1738	0.8792	0.123*	
C30B	1.2156 (4)	0.17911 (8)	0.8962 (2)	0.0840 (11)	
H30B	1.2182	0.1977	0.9007	0.101*	
C31B	0.7199 (3)	0.19342 (7)	1.01871 (17)	0.0590 (8)	
C32B	0.5990 (4)	0.20932 (7)	1.00437 (19)	0.0739 (10)	
H32B	0.576	0.2191	0.9615	0.089*	
C33B	0.5121 (4)	0.21075 (8)	1.0533 (2)	0.0851 (11)	
H33B	0.4304	0.2215	1.0432	0.102*	
C34B	0.5453 (4)	0.19646 (8)	1.1167 (2)	0.0789 (11)	
H34B	0.4866	0.1975	1.1498	0.095*	
C35B	0.6651 (5)	0.18076 (9)	1.1310 (2)	0.0888 (12)	
H35B	0.6878	0.1711	1.1741	0.107*	
C36B	0.7530 (4)	0.17897 (8)	1.08248 (19)	0.0826 (11)	
H36B	0.8341	0.1681	1.0927	0.099*	
C37B	0.9353 (4)	0.21091 (8)	0.81351 (17)	0.0656 (9)	
C38B	0.9292 (4)	0.19484 (8)	0.75467 (19)	0.0820 (11)	
H38B	0.9093	0.1766	0.7567	0.098*	
C39B	0.9533 (5)	0.20622 (10)	0.6918 (2)	0.0971 (13)	
H39B	0.9494	0.1956	0.6508	0.117*	
C40B	0.9828 (5)	0.23300 (11)	0.6900 (2)	0.1014 (15)	
H40B	0.9997	0.2405	0.6477	0.122*	
C41B	0.9880 (5)	0.24903 (10)	0.7497 (2)	0.0988 (14)	
H41B	1.0075	0.2673	0.7476	0.119*	
C42B	0.9641 (4)	0.23788 (9)	0.8132 (2)	0.0839 (11)	
H42B	0.9676	0.2484	0.8542	0.101*	

### Atomic displacement parameters (Å^2^)

**Table:**

	*U*^11^	*U*^22^	*U*^33^	*U*^12^	*U*^13^	*U*^23^
Co1A	0.0983 (4)	0.0674 (3)	0.0631 (3)	−0.0040 (3)	0.0238 (3)	0.0028 (2)
N1A	0.137 (3)	0.092 (3)	0.091 (2)	−0.021 (2)	0.045 (2)	−0.005 (2)
N2A	0.111 (3)	0.079 (2)	0.068 (2)	0.004 (2)	0.0123 (19)	0.0001 (18)
N3A	0.104 (3)	0.084 (2)	0.074 (2)	0.004 (2)	0.0253 (19)	0.0041 (18)
N4A	0.100 (3)	0.085 (2)	0.073 (2)	−0.003 (2)	0.0162 (19)	0.0013 (18)
N5A	0.0630 (19)	0.0620 (18)	0.0545 (16)	0.0062 (15)	0.0074 (14)	−0.0025 (14)
N6A	0.070 (2)	0.0643 (18)	0.0575 (16)	0.0066 (16)	0.0137 (15)	−0.0017 (14)
N7A	0.0596 (19)	0.078 (2)	0.0608 (17)	0.0043 (15)	0.0108 (15)	−0.0034 (15)
N8A	0.0600 (19)	0.0654 (18)	0.0554 (16)	0.0060 (15)	0.0038 (14)	−0.0058 (14)
N9A	0.0607 (19)	0.0639 (18)	0.0611 (17)	−0.0068 (15)	0.0144 (15)	−0.0007 (15)
N10A	0.0587 (18)	0.0671 (19)	0.0596 (17)	−0.0031 (15)	0.0133 (15)	0.0001 (14)
N11A	0.0577 (19)	0.074 (2)	0.0590 (17)	−0.0040 (15)	0.0090 (14)	−0.0011 (15)
N12A	0.0589 (19)	0.0721 (19)	0.0529 (16)	−0.0033 (15)	0.0065 (14)	0.0008 (15)
S1A	0.250 (2)	0.1215 (12)	0.1377 (12)	−0.0770 (13)	0.0892 (13)	0.0017 (10)
S2A	0.1284 (10)	0.0826 (7)	0.0715 (6)	0.0293 (7)	−0.0026 (6)	−0.0077 (6)
S3A	0.0828 (7)	0.0959 (8)	0.0944 (7)	0.0028 (6)	0.0313 (6)	−0.0060 (6)
S4A	0.0939 (8)	0.0746 (7)	0.0679 (6)	0.0080 (6)	0.0126 (5)	0.0009 (5)
C1A	0.110 (3)	0.088 (3)	0.067 (2)	−0.012 (3)	0.036 (2)	−0.004 (2)
C2A	0.083 (3)	0.072 (3)	0.064 (2)	0.010 (2)	0.009 (2)	0.012 (2)
C3A	0.075 (3)	0.079 (3)	0.063 (2)	−0.007 (2)	0.0194 (19)	0.003 (2)
C4A	0.070 (2)	0.068 (2)	0.064 (2)	−0.0022 (19)	0.0166 (19)	−0.0086 (19)
C5A	0.064 (2)	0.062 (2)	0.056 (2)	0.0100 (19)	0.0094 (18)	−0.0015 (17)
C6A	0.062 (2)	0.061 (2)	0.058 (2)	0.0050 (17)	0.0082 (18)	0.0022 (17)
C7A	0.074 (3)	0.101 (3)	0.059 (2)	−0.006 (2)	0.006 (2)	0.000 (2)
C8A	0.078 (3)	0.122 (4)	0.074 (3)	−0.013 (3)	0.005 (2)	−0.003 (2)
C9A	0.073 (3)	0.085 (3)	0.092 (3)	−0.006 (2)	0.015 (2)	0.001 (2)
C10A	0.091 (3)	0.077 (3)	0.082 (3)	−0.004 (2)	0.031 (3)	0.009 (2)
C11A	0.087 (3)	0.071 (2)	0.056 (2)	0.004 (2)	0.0047 (19)	0.0074 (18)
C12A	0.066 (2)	0.076 (3)	0.064 (2)	0.004 (2)	0.0126 (19)	−0.0077 (19)
C13A	0.111 (4)	0.092 (3)	0.091 (3)	−0.015 (3)	0.046 (3)	−0.021 (2)
C14A	0.133 (4)	0.114 (4)	0.121 (4)	−0.033 (3)	0.063 (3)	−0.023 (3)
C15A	0.119 (4)	0.135 (4)	0.091 (3)	−0.013 (3)	0.049 (3)	−0.008 (3)
C16A	0.102 (4)	0.141 (4)	0.075 (3)	−0.005 (3)	0.030 (3)	−0.027 (3)
C17A	0.082 (3)	0.108 (3)	0.070 (2)	−0.008 (2)	0.019 (2)	−0.020 (2)
C18A	0.068 (2)	0.061 (2)	0.054 (2)	0.0050 (18)	0.0126 (18)	−0.0080 (16)
C19A	0.078 (3)	0.080 (3)	0.060 (2)	0.021 (2)	0.0126 (19)	0.0028 (19)
C20A	0.092 (3)	0.079 (3)	0.080 (3)	0.024 (2)	0.029 (2)	−0.002 (2)
C21A	0.106 (3)	0.076 (3)	0.071 (3)	0.008 (2)	0.032 (2)	−0.011 (2)
C22A	0.108 (4)	0.116 (4)	0.056 (2)	0.021 (3)	0.004 (2)	−0.021 (2)
C23A	0.078 (3)	0.108 (3)	0.063 (2)	0.025 (2)	−0.002 (2)	−0.016 (2)
C24A	0.056 (2)	0.066 (2)	0.0537 (19)	−0.0020 (18)	0.0136 (17)	0.0013 (17)
C25A	0.055 (2)	0.074 (3)	0.063 (2)	−0.0093 (19)	0.0127 (17)	−0.0063 (18)
C26A	0.076 (3)	0.069 (3)	0.072 (2)	−0.007 (2)	0.022 (2)	−0.0093 (19)
C27A	0.090 (3)	0.071 (3)	0.093 (3)	−0.023 (3)	0.026 (2)	−0.020 (2)
C28A	0.080 (3)	0.100 (4)	0.111 (3)	−0.032 (3)	0.019 (3)	−0.019 (3)
C29A	0.064 (3)	0.097 (4)	0.148 (4)	−0.011 (3)	0.019 (3)	−0.012 (3)
C30A	0.065 (3)	0.076 (3)	0.121 (3)	−0.009 (2)	0.018 (2)	−0.011 (2)
C31A	0.053 (2)	0.069 (2)	0.058 (2)	−0.0019 (18)	0.0112 (17)	0.0011 (18)
C32A	0.068 (3)	0.088 (3)	0.103 (3)	−0.015 (2)	−0.004 (2)	0.030 (2)
C33A	0.077 (3)	0.108 (4)	0.140 (4)	−0.031 (3)	−0.011 (3)	0.037 (3)
C34A	0.064 (3)	0.121 (4)	0.101 (3)	−0.012 (3)	−0.004 (2)	0.013 (3)
C35A	0.082 (3)	0.112 (4)	0.082 (3)	−0.002 (3)	−0.003 (2)	0.022 (3)
C36A	0.078 (3)	0.096 (3)	0.075 (2)	−0.014 (2)	0.003 (2)	0.017 (2)
C37A	0.059 (2)	0.081 (3)	0.057 (2)	−0.0054 (19)	0.0047 (17)	0.000 (2)
C38A	0.089 (3)	0.102 (3)	0.074 (3)	0.014 (2)	−0.001 (2)	−0.009 (2)
C39A	0.106 (4)	0.140 (5)	0.071 (3)	0.014 (3)	−0.008 (3)	−0.012 (3)
C40A	0.086 (3)	0.137 (5)	0.082 (3)	0.001 (3)	−0.008 (3)	0.026 (3)
C41A	0.090 (3)	0.093 (3)	0.105 (4)	−0.004 (3)	−0.004 (3)	0.022 (3)
C42A	0.087 (3)	0.082 (3)	0.074 (3)	−0.011 (2)	0.001 (2)	0.008 (2)
Co1B	0.0868 (4)	0.0672 (3)	0.0604 (3)	0.0101 (3)	0.0031 (3)	0.0014 (2)
N1B	0.120 (3)	0.103 (3)	0.089 (2)	0.030 (2)	−0.009 (2)	−0.008 (2)
N2B	0.103 (3)	0.080 (2)	0.069 (2)	0.006 (2)	0.0078 (18)	−0.0011 (18)
N3B	0.108 (3)	0.081 (2)	0.067 (2)	0.002 (2)	0.0103 (19)	0.0041 (18)
N4B	0.090 (2)	0.086 (2)	0.071 (2)	0.0069 (19)	0.0134 (18)	0.0060 (18)
N5B	0.071 (2)	0.0558 (17)	0.0510 (15)	−0.0032 (15)	0.0142 (15)	−0.0012 (13)
N6B	0.0683 (19)	0.0635 (18)	0.0525 (16)	−0.0066 (16)	0.0097 (15)	−0.0006 (14)
N7B	0.069 (2)	0.0621 (18)	0.0598 (17)	−0.0053 (15)	0.0155 (15)	−0.0005 (14)
N8B	0.065 (2)	0.0613 (18)	0.0559 (16)	−0.0046 (15)	0.0129 (15)	−0.0023 (14)
N9B	0.0569 (18)	0.0703 (19)	0.0597 (17)	0.0033 (15)	0.0155 (14)	−0.0014 (15)
N10B	0.0588 (18)	0.0692 (18)	0.0574 (16)	0.0035 (15)	0.0177 (14)	−0.0003 (14)
N11B	0.0608 (18)	0.0735 (19)	0.0576 (16)	0.0035 (15)	0.0166 (14)	0.0014 (15)
N12B	0.0610 (18)	0.0684 (19)	0.0568 (16)	0.0017 (15)	0.0143 (14)	0.0027 (15)
S1B	0.1875 (17)	0.1201 (12)	0.1735 (15)	0.0766 (12)	−0.0467 (13)	−0.0115 (11)
S2B	0.1123 (9)	0.0803 (7)	0.0672 (6)	−0.0161 (6)	0.0171 (6)	−0.0078 (5)
S3B	0.0859 (8)	0.0977 (8)	0.0909 (7)	−0.0071 (6)	0.0119 (6)	−0.0065 (6)
S4B	0.0920 (7)	0.0664 (6)	0.0718 (6)	0.0033 (5)	0.0216 (5)	0.0057 (5)
C1B	0.082 (3)	0.080 (3)	0.076 (3)	0.016 (2)	−0.002 (2)	−0.009 (2)
C2B	0.077 (3)	0.069 (2)	0.060 (2)	0.004 (2)	0.0164 (19)	0.0085 (19)
C3B	0.076 (3)	0.075 (3)	0.056 (2)	0.013 (2)	0.0111 (19)	0.006 (2)
C4B	0.064 (2)	0.067 (2)	0.063 (2)	0.0008 (18)	0.0148 (18)	−0.0073 (19)
C5B	0.067 (2)	0.058 (2)	0.054 (2)	−0.0065 (18)	0.0158 (18)	0.0026 (17)
C6B	0.064 (2)	0.056 (2)	0.060 (2)	−0.0010 (17)	0.0122 (18)	0.0007 (16)
C7B	0.081 (3)	0.080 (3)	0.061 (2)	0.004 (2)	0.019 (2)	0.0024 (19)
C8B	0.092 (3)	0.093 (3)	0.083 (3)	0.013 (3)	0.036 (3)	0.004 (2)
C9B	0.080 (3)	0.074 (3)	0.100 (3)	0.006 (2)	0.021 (3)	0.004 (2)
C10B	0.092 (3)	0.080 (3)	0.074 (3)	0.012 (2)	0.005 (2)	0.006 (2)
C11B	0.091 (3)	0.074 (3)	0.062 (2)	0.008 (2)	0.017 (2)	0.0044 (19)
C12B	0.065 (2)	0.072 (2)	0.061 (2)	−0.007 (2)	0.0122 (19)	0.0070 (19)
C13B	0.076 (3)	0.075 (3)	0.084 (3)	−0.005 (2)	0.013 (2)	0.003 (2)
C14B	0.077 (3)	0.093 (3)	0.109 (3)	0.003 (2)	0.011 (3)	0.015 (3)
C15B	0.082 (3)	0.116 (4)	0.088 (3)	−0.010 (3)	−0.006 (3)	0.024 (3)
C16B	0.098 (4)	0.130 (4)	0.070 (3)	−0.003 (3)	−0.001 (3)	−0.006 (3)
C17B	0.082 (3)	0.104 (3)	0.064 (2)	−0.001 (2)	0.009 (2)	−0.004 (2)
C18B	0.072 (2)	0.060 (2)	0.056 (2)	−0.0015 (19)	0.0019 (18)	−0.0054 (17)
C19B	0.081 (3)	0.079 (3)	0.083 (3)	−0.013 (2)	0.005 (2)	0.002 (2)
C20B	0.088 (3)	0.090 (3)	0.108 (4)	−0.016 (2)	−0.012 (3)	−0.004 (3)
C21B	0.125 (4)	0.084 (3)	0.093 (4)	−0.001 (3)	−0.018 (3)	−0.026 (3)
C22B	0.143 (5)	0.128 (4)	0.079 (3)	−0.015 (4)	0.015 (3)	−0.037 (3)
C23B	0.105 (3)	0.114 (3)	0.073 (3)	−0.021 (3)	0.026 (2)	−0.030 (2)
C24B	0.057 (2)	0.068 (2)	0.0524 (19)	0.0043 (18)	0.0095 (16)	0.0021 (17)
C25B	0.059 (2)	0.073 (3)	0.063 (2)	0.0041 (19)	0.0189 (17)	−0.0096 (18)
C26B	0.069 (3)	0.080 (3)	0.070 (2)	0.001 (2)	0.0170 (19)	−0.007 (2)
C27B	0.090 (3)	0.078 (3)	0.091 (3)	0.011 (2)	0.026 (2)	−0.012 (2)
C28B	0.084 (3)	0.102 (4)	0.124 (4)	0.012 (3)	0.045 (3)	−0.021 (3)
C29B	0.072 (3)	0.101 (4)	0.143 (4)	−0.008 (3)	0.046 (3)	−0.026 (3)
C30B	0.069 (3)	0.082 (3)	0.109 (3)	−0.003 (2)	0.036 (2)	−0.017 (2)
C31B	0.055 (2)	0.068 (2)	0.0546 (19)	0.0042 (17)	0.0135 (16)	−0.0006 (17)
C32B	0.071 (2)	0.086 (3)	0.069 (2)	0.018 (2)	0.023 (2)	0.014 (2)
C33B	0.067 (3)	0.098 (3)	0.095 (3)	0.021 (2)	0.027 (2)	0.004 (2)
C34B	0.077 (3)	0.087 (3)	0.081 (3)	−0.005 (2)	0.036 (2)	−0.009 (2)
C35B	0.097 (3)	0.110 (3)	0.065 (2)	0.018 (3)	0.030 (2)	0.021 (2)
C36B	0.081 (3)	0.105 (3)	0.066 (2)	0.026 (2)	0.026 (2)	0.017 (2)
C37B	0.067 (2)	0.076 (3)	0.056 (2)	−0.0095 (19)	0.0185 (17)	0.0052 (19)
C38B	0.103 (3)	0.084 (3)	0.066 (2)	−0.014 (2)	0.033 (2)	−0.007 (2)
C39B	0.120 (4)	0.112 (4)	0.067 (3)	−0.022 (3)	0.036 (2)	−0.007 (2)
C40B	0.104 (3)	0.133 (4)	0.072 (3)	−0.034 (3)	0.031 (2)	0.012 (3)
C41B	0.106 (3)	0.097 (3)	0.092 (3)	−0.034 (3)	0.019 (3)	0.015 (3)
C42B	0.089 (3)	0.091 (3)	0.069 (2)	−0.023 (2)	0.013 (2)	0.000 (2)

### Geometric parameters (Å, °)

**Table:**

Co1A—N1A	1.940 (4)	Co1B—N1B	1.942 (4)
Co1A—N2A	1.942 (4)	Co1B—N2B	1.950 (3)
Co1A—N3A	1.958 (4)	Co1B—N3B	1.953 (4)
Co1A—N4A	1.951 (4)	Co1B—N4B	1.944 (3)
N1A—C1A	1.154 (4)	N1B—C1B	1.144 (4)
N2A—C2A	1.160 (4)	N2B—C2B	1.164 (4)
N3A—C3A	1.167 (4)	N3B—C3B	1.168 (4)
N4A—C4A	1.167 (4)	N4B—C4B	1.164 (4)
N5A—N6A	1.319 (3)	N5B—N6B	1.311 (3)
N5A—N8A	1.344 (3)	N5B—N8B	1.348 (3)
N5A—C6A	1.443 (4)	N5B—C6B	1.443 (4)
N6A—C5A	1.343 (4)	N6B—C5B	1.350 (4)
N7A—N8A	1.317 (3)	N7B—N8B	1.312 (3)
N7A—C5A	1.353 (4)	N7B—C5B	1.343 (4)
N8A—C18A	1.455 (4)	N8B—C18B	1.444 (4)
N9A—N10A	1.317 (3)	N9B—N10B	1.313 (3)
N9A—N12A	1.341 (3)	N9B—N12B	1.335 (3)
N9A—C25A	1.448 (4)	N9B—C25B	1.447 (4)
N10A—C24A	1.351 (4)	N10B—C24B	1.344 (4)
N11A—N12A	1.314 (3)	N11B—N12B	1.319 (3)
N11A—C24A	1.342 (4)	N11B—C24B	1.350 (4)
N12A—C37A	1.446 (4)	N12B—C37B	1.451 (4)
S1A—C1A	1.575 (5)	S1B—C1B	1.566 (4)
S2A—C2A	1.615 (4)	S2B—C2B	1.606 (4)
S3A—C3A	1.608 (4)	S3B—C3B	1.608 (5)
S4A—C4A	1.603 (4)	S4B—C4B	1.603 (4)
C5A—C12A	1.453 (5)	C5B—C12B	1.457 (5)
C6A—C11A	1.368 (4)	C6B—C11B	1.371 (4)
C6A—C7A	1.374 (5)	C6B—C7B	1.377 (4)
C7A—C8A	1.378 (5)	C7B—C8B	1.378 (5)
C7A—H7A	0.93	C7B—H7B	0.93
C8A—C9A	1.372 (5)	C8B—C9B	1.368 (5)
C8A—H8A	0.93	C8B—H8B	0.93
C9A—C10A	1.360 (5)	C9B—C10B	1.372 (5)
C9A—H9A	0.93	C9B—H9B	0.93
C10A—C11A	1.389 (5)	C10B—C11B	1.384 (5)
C10A—H10A	0.93	C10B—H10B	0.93
C11A—H11A	0.93	C11B—H11B	0.93
C12A—C13A	1.380 (5)	C12B—C17B	1.385 (5)
C12A—C17A	1.391 (5)	C12B—C13B	1.387 (5)
C13A—C14A	1.381 (5)	C13B—C14B	1.381 (5)
C13A—H13A	0.93	C13B—H13B	0.93
C14A—C15A	1.373 (6)	C14B—C15B	1.372 (6)
C14A—H14A	0.93	C14B—H14B	0.93
C15A—C16A	1.367 (6)	C15B—C16B	1.376 (6)
C15A—H15A	0.93	C15B—H15B	0.93
C16A—C17A	1.376 (5)	C16B—C17B	1.372 (5)
C16A—H16A	0.93	C16B—H16B	0.93
C17A—H17A	0.93	C17B—H17B	0.93
C18A—C23A	1.366 (4)	C18B—C19B	1.365 (5)
C18A—C19A	1.367 (4)	C18B—C23B	1.376 (5)
C19A—C20A	1.376 (5)	C19B—C20B	1.398 (5)
C19A—H19A	0.93	C19B—H19B	0.93
C20A—C21A	1.367 (5)	C20B—C21B	1.372 (6)
C20A—H20A	0.93	C20B—H20B	0.93
C21A—C22A	1.360 (5)	C21B—C22B	1.347 (6)
C21A—H21A	0.93	C21B—H21B	0.93
C22A—C23A	1.384 (5)	C22B—C23B	1.373 (6)
C22A—H22A	0.93	C22B—H22B	0.93
C23A—H23A	0.93	C23B—H23B	0.93
C24A—C31A	1.452 (4)	C24B—C31B	1.457 (4)
C25A—C26A	1.366 (5)	C25B—C26B	1.369 (5)
C25A—C30A	1.372 (5)	C25B—C30B	1.371 (5)
C26A—C27A	1.383 (5)	C26B—C27B	1.381 (5)
C26A—H26A	0.93	C26B—H26B	0.93
C27A—C28A	1.372 (5)	C27B—C28B	1.365 (5)
C27A—H27A	0.93	C27B—H27B	0.93
C28A—C29A	1.368 (6)	C28B—C29B	1.366 (6)
C28A—H28A	0.93	C28B—H28B	0.93
C29A—C30A	1.388 (5)	C29B—C30B	1.388 (5)
C29A—H29A	0.93	C29B—H29B	0.93
C30A—H30A	0.93	C30B—H30B	0.93
C31A—C36A	1.370 (5)	C31B—C32B	1.378 (4)
C31A—C32A	1.374 (5)	C31B—C36B	1.379 (4)
C32A—C33A	1.375 (5)	C32B—C33B	1.375 (5)
C32A—H32A	0.93	C32B—H32B	0.93
C33A—C34A	1.363 (6)	C33B—C34B	1.369 (5)
C33A—H33A	0.93	C33B—H33B	0.93
C34A—C35A	1.354 (5)	C34B—C35B	1.363 (5)
C34A—H34A	0.93	C34B—H34B	0.93
C35A—C36A	1.378 (5)	C35B—C36B	1.377 (5)
C35A—H35A	0.93	C35B—H35B	0.93
C36A—H36A	0.93	C36B—H36B	0.93
C37A—C38A	1.361 (5)	C37B—C38B	1.360 (5)
C37A—C42A	1.371 (5)	C37B—C42B	1.369 (5)
C38A—C39A	1.379 (6)	C38B—C39B	1.380 (5)
C38A—H38A	0.93	C38B—H38B	0.93
C39A—C40A	1.361 (6)	C39B—C40B	1.363 (6)
C39A—H39A	0.93	C39B—H39B	0.93
C40A—C41A	1.369 (6)	C40B—C41B	1.373 (6)
C40A—H40A	0.93	C40B—H40B	0.93
C41A—C42A	1.385 (5)	C41B—C42B	1.385 (5)
C41A—H41A	0.93	C41B—H41B	0.93
C42A—H42A	0.93	C42B—H42B	0.93
			
N1A—Co1A—N2A	106.23 (14)	N1B—Co1B—N2B	103.04 (14)
N1A—Co1A—N3A	103.62 (15)	N1B—Co1B—N3B	105.05 (16)
N1A—Co1A—N4A	114.03 (16)	N1B—Co1B—N4B	117.79 (15)
N2A—Co1A—N3A	117.86 (14)	N2B—Co1B—N3B	120.13 (14)
N2A—Co1A—N4A	107.62 (13)	N2B—Co1B—N4B	106.41 (13)
N3A—Co1A—N4A	107.73 (13)	N3B—Co1B—N4B	105.23 (13)
C1A—N1A—Co1A	162.9 (4)	C1B—N1B—Co1B	161.7 (4)
C2A—N2A—Co1A	165.2 (4)	C2B—N2B—Co1B	165.4 (3)
C3A—N3A—Co1A	163.7 (3)	C3B—N3B—Co1B	161.0 (3)
C4A—N4A—Co1A	172.6 (3)	C4B—N4B—Co1B	171.2 (3)
N6A—N5A—N8A	109.3 (3)	N6B—N5B—N8B	109.5 (3)
N6A—N5A—C6A	122.6 (3)	N6B—N5B—C6B	123.7 (3)
N8A—N5A—C6A	127.9 (3)	N8B—N5B—C6B	126.8 (3)
N5A—N6A—C5A	104.7 (3)	N5B—N6B—C5B	104.3 (3)
N8A—N7A—C5A	104.0 (3)	N8B—N7B—C5B	104.2 (3)
N7A—N8A—N5A	110.1 (3)	N7B—N8B—N5B	109.9 (3)
N7A—N8A—C18A	123.4 (3)	N7B—N8B—C18B	122.7 (3)
N5A—N8A—C18A	126.4 (3)	N5B—N8B—C18B	127.2 (3)
N10A—N9A—N12A	109.6 (3)	N10B—N9B—N12B	110.2 (3)
N10A—N9A—C25A	123.7 (3)	N10B—N9B—C25B	123.3 (3)
N12A—N9A—C25A	126.6 (3)	N12B—N9B—C25B	126.5 (3)
N9A—N10A—C24A	104.0 (3)	N9B—N10B—C24B	103.7 (3)
N12A—N11A—C24A	104.0 (3)	N12B—N11B—C24B	103.3 (3)
N11A—N12A—N9A	110.1 (3)	N11B—N12B—N9B	110.1 (2)
N11A—N12A—C37A	122.7 (3)	N11B—N12B—C37B	123.6 (3)
N9A—N12A—C37A	127.1 (3)	N9B—N12B—C37B	126.2 (3)
N1A—C1A—S1A	179.5 (5)	N1B—C1B—S1B	179.4 (4)
N2A—C2A—S2A	179.9 (4)	N2B—C2B—S2B	178.2 (4)
N3A—C3A—S3A	179.5 (4)	N3B—C3B—S3B	179.3 (4)
N4A—C4A—S4A	178.9 (3)	N4B—C4B—S4B	178.7 (4)
N6A—C5A—N7A	111.9 (3)	N7B—C5B—N6B	112.0 (3)
N6A—C5A—C12A	123.7 (3)	N7B—C5B—C12B	123.7 (3)
N7A—C5A—C12A	124.3 (3)	N6B—C5B—C12B	124.2 (3)
C11A—C6A—C7A	122.5 (4)	C11B—C6B—C7B	122.2 (3)
C11A—C6A—N5A	121.3 (3)	C11B—C6B—N5B	120.8 (3)
C7A—C6A—N5A	116.3 (3)	C7B—C6B—N5B	117.0 (3)
C6A—C7A—C8A	118.1 (4)	C6B—C7B—C8B	118.2 (4)
C6A—C7A—H7A	121	C6B—C7B—H7B	120.9
C8A—C7A—H7A	121	C8B—C7B—H7B	120.9
C9A—C8A—C7A	120.8 (4)	C9B—C8B—C7B	120.8 (4)
C9A—C8A—H8A	119.6	C9B—C8B—H8B	119.6
C7A—C8A—H8A	119.6	C7B—C8B—H8B	119.6
C10A—C9A—C8A	119.9 (4)	C8B—C9B—C10B	120.0 (4)
C10A—C9A—H9A	120	C8B—C9B—H9B	120
C8A—C9A—H9A	120	C10B—C9B—H9B	120
C9A—C10A—C11A	120.9 (4)	C9B—C10B—C11B	120.5 (4)
C9A—C10A—H10A	119.6	C9B—C10B—H10B	119.7
C11A—C10A—H10A	119.6	C11B—C10B—H10B	119.7
C6A—C11A—C10A	117.8 (3)	C6B—C11B—C10B	118.2 (3)
C6A—C11A—H11A	121.1	C6B—C11B—H11B	120.9
C10A—C11A—H11A	121.1	C10B—C11B—H11B	120.9
C13A—C12A—C17A	119.0 (4)	C17B—C12B—C13B	119.2 (4)
C13A—C12A—C5A	121.0 (3)	C17B—C12B—C5B	120.6 (4)
C17A—C12A—C5A	119.9 (4)	C13B—C12B—C5B	120.2 (3)
C12A—C13A—C14A	120.2 (4)	C14B—C13B—C12B	120.2 (4)
C12A—C13A—H13A	119.9	C14B—C13B—H13B	119.9
C14A—C13A—H13A	119.9	C12B—C13B—H13B	119.9
C15A—C14A—C13A	120.1 (5)	C15B—C14B—C13B	119.7 (4)
C15A—C14A—H14A	119.9	C15B—C14B—H14B	120.1
C13A—C14A—H14A	119.9	C13B—C14B—H14B	120.1
C16A—C15A—C14A	120.1 (4)	C14B—C15B—C16B	120.5 (4)
C16A—C15A—H15A	120	C14B—C15B—H15B	119.8
C14A—C15A—H15A	120	C16B—C15B—H15B	119.8
C15A—C16A—C17A	120.3 (4)	C17B—C16B—C15B	120.1 (4)
C15A—C16A—H16A	119.8	C17B—C16B—H16B	120
C17A—C16A—H16A	119.8	C15B—C16B—H16B	120
C16A—C17A—C12A	120.2 (4)	C16B—C17B—C12B	120.3 (4)
C16A—C17A—H17A	119.9	C16B—C17B—H17B	119.9
C12A—C17A—H17A	119.9	C12B—C17B—H17B	119.9
C23A—C18A—C19A	122.9 (3)	C19B—C18B—C23B	123.4 (4)
C23A—C18A—N8A	118.4 (3)	C19B—C18B—N8B	118.9 (3)
C19A—C18A—N8A	118.7 (3)	C23B—C18B—N8B	117.5 (3)
C18A—C19A—C20A	117.9 (3)	C18B—C19B—C20B	117.3 (4)
C18A—C19A—H19A	121	C18B—C19B—H19B	121.4
C20A—C19A—H19A	121	C20B—C19B—H19B	121.4
C21A—C20A—C19A	120.7 (4)	C21B—C20B—C19B	119.7 (4)
C21A—C20A—H20A	119.7	C21B—C20B—H20B	120.2
C19A—C20A—H20A	119.7	C19B—C20B—H20B	120.2
C22A—C21A—C20A	120.0 (4)	C22B—C21B—C20B	121.1 (4)
C22A—C21A—H21A	120	C22B—C21B—H21B	119.5
C20A—C21A—H21A	120	C20B—C21B—H21B	119.5
C21A—C22A—C23A	120.9 (4)	C21B—C22B—C23B	121.1 (5)
C21A—C22A—H22A	119.5	C21B—C22B—H22B	119.4
C23A—C22A—H22A	119.5	C23B—C22B—H22B	119.4
C18A—C23A—C22A	117.5 (4)	C22B—C23B—C18B	117.4 (4)
C18A—C23A—H23A	121.2	C22B—C23B—H23B	121.3
C22A—C23A—H23A	121.2	C18B—C23B—H23B	121.3
N11A—C24A—N10A	112.2 (3)	N10B—C24B—N11B	112.7 (3)
N11A—C24A—C31A	123.1 (3)	N10B—C24B—C31B	123.9 (3)
N10A—C24A—C31A	124.7 (3)	N11B—C24B—C31B	123.5 (3)
C26A—C25A—C30A	123.9 (3)	C26B—C25B—C30B	123.7 (3)
C26A—C25A—N9A	117.4 (3)	C26B—C25B—N9B	117.6 (3)
C30A—C25A—N9A	118.6 (3)	C30B—C25B—N9B	118.6 (3)
C25A—C26A—C27A	117.4 (4)	C25B—C26B—C27B	117.9 (4)
C25A—C26A—H26A	121.3	C25B—C26B—H26B	121
C27A—C26A—H26A	121.3	C27B—C26B—H26B	121
C28A—C27A—C26A	120.4 (4)	C28B—C27B—C26B	119.9 (4)
C28A—C27A—H27A	119.8	C28B—C27B—H27B	120
C26A—C27A—H27A	119.8	C26B—C27B—H27B	120
C29A—C28A—C27A	120.8 (4)	C27B—C28B—C29B	121.1 (4)
C29A—C28A—H28A	119.6	C27B—C28B—H28B	119.5
C27A—C28A—H28A	119.6	C29B—C28B—H28B	119.5
C28A—C29A—C30A	120.3 (4)	C28B—C29B—C30B	120.5 (4)
C28A—C29A—H29A	119.9	C28B—C29B—H29B	119.7
C30A—C29A—H29A	119.9	C30B—C29B—H29B	119.7
C25A—C30A—C29A	117.3 (4)	C25B—C30B—C29B	116.8 (4)
C25A—C30A—H30A	121.4	C25B—C30B—H30B	121.6
C29A—C30A—H30A	121.4	C29B—C30B—H30B	121.6
C36A—C31A—C32A	119.1 (3)	C32B—C31B—C36B	119.4 (3)
C36A—C31A—C24A	121.2 (3)	C32B—C31B—C24B	121.1 (3)
C32A—C31A—C24A	119.7 (3)	C36B—C31B—C24B	119.5 (3)
C31A—C32A—C33A	120.0 (4)	C33B—C32B—C31B	120.3 (3)
C31A—C32A—H32A	120	C33B—C32B—H32B	119.9
C33A—C32A—H32A	120	C31B—C32B—H32B	119.9
C34A—C33A—C32A	120.5 (4)	C34B—C33B—C32B	120.3 (4)
C34A—C33A—H33A	119.8	C34B—C33B—H33B	119.8
C32A—C33A—H33A	119.8	C32B—C33B—H33B	119.8
C35A—C34A—C33A	119.7 (4)	C35B—C34B—C33B	119.5 (3)
C35A—C34A—H34A	120.2	C35B—C34B—H34B	120.3
C33A—C34A—H34A	120.2	C33B—C34B—H34B	120.3
C34A—C35A—C36A	120.6 (4)	C34B—C35B—C36B	121.1 (4)
C34A—C35A—H35A	119.7	C34B—C35B—H35B	119.5
C36A—C35A—H35A	119.7	C36B—C35B—H35B	119.5
C31A—C36A—C35A	120.1 (4)	C35B—C36B—C31B	119.5 (4)
C31A—C36A—H36A	120	C35B—C36B—H36B	120.3
C35A—C36A—H36A	120	C31B—C36B—H36B	120.3
C38A—C37A—C42A	122.6 (4)	C38B—C37B—C42B	122.9 (3)
C38A—C37A—N12A	120.1 (4)	C38B—C37B—N12B	119.3 (3)
C42A—C37A—N12A	117.2 (3)	C42B—C37B—N12B	117.8 (3)
C37A—C38A—C39A	118.1 (4)	C37B—C38B—C39B	118.6 (4)
C37A—C38A—H38A	120.9	C37B—C38B—H38B	120.7
C39A—C38A—H38A	120.9	C39B—C38B—H38B	120.7
C40A—C39A—C38A	120.6 (4)	C40B—C39B—C38B	119.8 (4)
C40A—C39A—H39A	119.7	C40B—C39B—H39B	120.1
C38A—C39A—H39A	119.7	C38B—C39B—H39B	120.1
C39A—C40A—C41A	120.5 (4)	C39B—C40B—C41B	121.1 (4)
C39A—C40A—H40A	119.7	C39B—C40B—H40B	119.5
C41A—C40A—H40A	119.7	C41B—C40B—H40B	119.5
C40A—C41A—C42A	119.9 (4)	C40B—C41B—C42B	119.7 (4)
C40A—C41A—H41A	120.1	C40B—C41B—H41B	120.1
C42A—C41A—H41A	120.1	C42B—C41B—H41B	120.1
C37A—C42A—C41A	118.2 (4)	C37B—C42B—C41B	117.9 (4)
C37A—C42A—H42A	120.9	C37B—C42B—H42B	121
C41A—C42A—H42A	120.9	C41B—C42B—H42B	121
			
N2A—Co1A—N1A—C1A	117.0 (12)	N4B—Co1B—N1B—C1B	−153.3 (11)
N4A—Co1A—N1A—C1A	−124.6 (12)	N2B—Co1B—N1B—C1B	90.0 (11)
N3A—Co1A—N1A—C1A	−7.8 (12)	N3B—Co1B—N1B—C1B	−36.6 (11)
N1A—Co1A—N2A—C2A	33.2 (12)	N1B—Co1B—N2B—C2B	46.2 (12)
N4A—Co1A—N2A—C2A	−89.3 (12)	N4B—Co1B—N2B—C2B	−78.3 (12)
N3A—Co1A—N2A—C2A	148.7 (12)	N3B—Co1B—N2B—C2B	162.5 (12)
N1A—Co1A—N3A—C3A	−37.8 (11)	N1B—Co1B—N3B—C3B	−52.0 (10)
N2A—Co1A—N3A—C3A	−154.7 (11)	N4B—Co1B—N3B—C3B	73.0 (10)
N4A—Co1A—N3A—C3A	83.4 (11)	N2B—Co1B—N3B—C3B	−167.2 (9)
N8A—N5A—N6A—C5A	0.1 (3)	N8B—N5B—N6B—C5B	−0.1 (3)
C6A—N5A—N6A—C5A	−175.7 (3)	C6B—N5B—N6B—C5B	−178.7 (3)
C5A—N7A—N8A—N5A	−1.4 (3)	C5B—N7B—N8B—N5B	−1.9 (3)
C5A—N7A—N8A—C18A	175.4 (3)	C5B—N7B—N8B—C18B	173.7 (3)
N6A—N5A—N8A—N7A	0.9 (3)	N6B—N5B—N8B—N7B	1.3 (3)
C6A—N5A—N8A—N7A	176.4 (3)	C6B—N5B—N8B—N7B	179.9 (3)
N6A—N5A—N8A—C18A	−175.8 (3)	N6B—N5B—N8B—C18B	−174.0 (3)
C6A—N5A—N8A—C18A	−0.3 (5)	C6B—N5B—N8B—C18B	4.6 (5)
N12A—N9A—N10A—C24A	−0.7 (3)	N12B—N9B—N10B—C24B	−1.0 (3)
C25A—N9A—N10A—C24A	176.2 (3)	C25B—N9B—N10B—C24B	177.5 (3)
C24A—N11A—N12A—N9A	0.1 (3)	C24B—N11B—N12B—N9B	−0.3 (3)
C24A—N11A—N12A—C37A	−179.7 (3)	C24B—N11B—N12B—C37B	178.1 (3)
N10A—N9A—N12A—N11A	0.4 (3)	N10B—N9B—N12B—N11B	0.8 (3)
C25A—N9A—N12A—N11A	−176.4 (3)	C25B—N9B—N12B—N11B	−177.6 (3)
N10A—N9A—N12A—C37A	−179.8 (3)	N10B—N9B—N12B—C37B	−177.5 (3)
C25A—N9A—N12A—C37A	3.3 (5)	C25B—N9B—N12B—C37B	4.0 (5)
N5A—N6A—C5A—N7A	−1.0 (4)	N8B—N7B—C5B—N6B	1.9 (3)
N5A—N6A—C5A—C12A	175.1 (3)	N8B—N7B—C5B—C12B	−175.6 (3)
N8A—N7A—C5A—N6A	1.5 (4)	N5B—N6B—C5B—N7B	−1.1 (3)
N8A—N7A—C5A—C12A	−174.5 (3)	N5B—N6B—C5B—C12B	176.3 (3)
N6A—N5A—C6A—C11A	−141.3 (3)	N6B—N5B—C6B—C11B	−141.5 (3)
N8A—N5A—C6A—C11A	43.8 (5)	N8B—N5B—C6B—C11B	40.1 (5)
N6A—N5A—C6A—C7A	37.6 (4)	N6B—N5B—C6B—C7B	37.0 (4)
N8A—N5A—C6A—C7A	−137.4 (3)	N8B—N5B—C6B—C7B	−141.4 (3)
C11A—C6A—C7A—C8A	−0.1 (6)	C11B—C6B—C7B—C8B	−1.4 (5)
N5A—C6A—C7A—C8A	−178.9 (3)	N5B—C6B—C7B—C8B	−179.9 (3)
C6A—C7A—C8A—C9A	−2.0 (6)	C6B—C7B—C8B—C9B	−1.4 (6)
C7A—C8A—C9A—C10A	2.3 (7)	C7B—C8B—C9B—C10B	2.3 (6)
C8A—C9A—C10A—C11A	−0.5 (6)	C8B—C9B—C10B—C11B	−0.6 (6)
C7A—C6A—C11A—C10A	1.8 (5)	C7B—C6B—C11B—C10B	3.1 (5)
N5A—C6A—C11A—C10A	−179.4 (3)	N5B—C6B—C11B—C10B	−178.5 (3)
C9A—C10A—C11A—C6A	−1.5 (6)	C9B—C10B—C11B—C6B	−2.1 (6)
N6A—C5A—C12A—C13A	−168.5 (4)	N7B—C5B—C12B—C17B	−178.1 (3)
N7A—C5A—C12A—C13A	7.1 (5)	N6B—C5B—C12B—C17B	4.7 (5)
N6A—C5A—C12A—C17A	7.9 (5)	N7B—C5B—C12B—C13B	4.0 (5)
N7A—C5A—C12A—C17A	−176.5 (3)	N6B—C5B—C12B—C13B	−173.1 (3)
C17A—C12A—C13A—C14A	0.2 (6)	C17B—C12B—C13B—C14B	0.1 (5)
C5A—C12A—C13A—C14A	176.7 (4)	C5B—C12B—C13B—C14B	178.0 (3)
C12A—C13A—C14A—C15A	−0.4 (8)	C12B—C13B—C14B—C15B	−0.2 (6)
C13A—C14A—C15A—C16A	1.0 (8)	C13B—C14B—C15B—C16B	0.4 (7)
C14A—C15A—C16A—C17A	−1.3 (8)	C14B—C15B—C16B—C17B	−0.5 (7)
C15A—C16A—C17A—C12A	1.0 (7)	C15B—C16B—C17B—C12B	0.4 (6)
C13A—C12A—C17A—C16A	−0.4 (6)	C13B—C12B—C17B—C16B	−0.2 (6)
C5A—C12A—C17A—C16A	−177.0 (4)	C5B—C12B—C17B—C16B	−178.1 (4)
N7A—N8A—C18A—C23A	64.0 (4)	N7B—N8B—C18B—C19B	−119.6 (4)
N5A—N8A—C18A—C23A	−119.8 (4)	N5B—N8B—C18B—C19B	55.1 (5)
N7A—N8A—C18A—C19A	−114.1 (4)	N7B—N8B—C18B—C23B	56.7 (4)
N5A—N8A—C18A—C19A	62.1 (4)	N5B—N8B—C18B—C23B	−128.5 (4)
C23A—C18A—C19A—C20A	−0.1 (6)	C23B—C18B—C19B—C20B	0.7 (6)
N8A—C18A—C19A—C20A	178.0 (3)	N8B—C18B—C19B—C20B	176.8 (3)
C18A—C19A—C20A—C21A	−0.2 (6)	C18B—C19B—C20B—C21B	−0.2 (6)
C19A—C20A—C21A—C22A	0.5 (6)	C19B—C20B—C21B—C22B	−0.8 (7)
C20A—C21A—C22A—C23A	−0.5 (7)	C20B—C21B—C22B—C23B	1.2 (8)
C19A—C18A—C23A—C22A	0.1 (6)	C21B—C22B—C23B—C18B	−0.7 (7)
N8A—C18A—C23A—C22A	−178.0 (4)	C19B—C18B—C23B—C22B	−0.2 (6)
C21A—C22A—C23A—C18A	0.2 (7)	N8B—C18B—C23B—C22B	−176.4 (4)
N12A—N11A—C24A—N10A	−0.5 (3)	N9B—N10B—C24B—N11B	0.9 (4)
N12A—N11A—C24A—C31A	177.0 (3)	N9B—N10B—C24B—C31B	−178.2 (3)
N9A—N10A—C24A—N11A	0.8 (3)	N12B—N11B—C24B—N10B	−0.4 (4)
N9A—N10A—C24A—C31A	−176.7 (3)	N12B—N11B—C24B—C31B	178.6 (3)
N10A—N9A—C25A—C26A	53.5 (4)	N10B—N9B—C25B—C26B	53.1 (4)
N12A—N9A—C25A—C26A	−130.1 (3)	N12B—N9B—C25B—C26B	−128.6 (3)
N10A—N9A—C25A—C30A	−122.4 (4)	N10B—N9B—C25B—C30B	−124.2 (4)
N12A—N9A—C25A—C30A	54.0 (5)	N12B—N9B—C25B—C30B	54.1 (5)
C30A—C25A—C26A—C27A	−1.2 (5)	C30B—C25B—C26B—C27B	−0.8 (5)
N9A—C25A—C26A—C27A	−176.9 (3)	N9B—C25B—C26B—C27B	−178.0 (3)
C25A—C26A—C27A—C28A	0.4 (6)	C25B—C26B—C27B—C28B	−0.5 (6)
C26A—C27A—C28A—C29A	0.0 (7)	C26B—C27B—C28B—C29B	1.3 (7)
C27A—C28A—C29A—C30A	0.3 (7)	C27B—C28B—C29B—C30B	−0.8 (7)
C26A—C25A—C30A—C29A	1.5 (6)	C26B—C25B—C30B—C29B	1.3 (6)
N9A—C25A—C30A—C29A	177.2 (3)	N9B—C25B—C30B—C29B	178.4 (3)
C28A—C29A—C30A—C25A	−1.0 (7)	C28B—C29B—C30B—C25B	−0.5 (7)
N11A—C24A—C31A—C36A	174.4 (3)	N10B—C24B—C31B—C32B	−168.3 (3)
N10A—C24A—C31A—C36A	−8.4 (5)	N11B—C24B—C31B—C32B	12.8 (5)
N11A—C24A—C31A—C32A	−5.9 (5)	N10B—C24B—C31B—C36B	10.8 (5)
N10A—C24A—C31A—C32A	171.3 (3)	N11B—C24B—C31B—C36B	−168.2 (3)
C36A—C31A—C32A—C33A	1.0 (6)	C36B—C31B—C32B—C33B	0.0 (6)
C24A—C31A—C32A—C33A	−178.7 (4)	C24B—C31B—C32B—C33B	179.0 (3)
C31A—C32A—C33A—C34A	0.3 (7)	C31B—C32B—C33B—C34B	0.2 (6)
C32A—C33A—C34A—C35A	−1.0 (8)	C32B—C33B—C34B—C35B	−0.1 (6)
C33A—C34A—C35A—C36A	0.4 (7)	C33B—C34B—C35B—C36B	−0.2 (6)
C32A—C31A—C36A—C35A	−1.5 (6)	C34B—C35B—C36B—C31B	0.4 (6)
C24A—C31A—C36A—C35A	178.2 (4)	C32B—C31B—C36B—C35B	−0.3 (6)
C34A—C35A—C36A—C31A	0.8 (7)	C24B—C31B—C36B—C35B	−179.4 (4)
N11A—N12A—C37A—C38A	−119.7 (4)	N11B—N12B—C37B—C38B	−121.1 (4)
N9A—N12A—C37A—C38A	60.6 (5)	N9B—N12B—C37B—C38B	57.0 (5)
N11A—N12A—C37A—C42A	56.4 (4)	N11B—N12B—C37B—C42B	57.1 (5)
N9A—N12A—C37A—C42A	−123.3 (4)	N9B—N12B—C37B—C42B	−124.8 (4)
C42A—C37A—C38A—C39A	−0.7 (6)	C42B—C37B—C38B—C39B	0.0 (6)
N12A—C37A—C38A—C39A	175.2 (4)	N12B—C37B—C38B—C39B	178.2 (4)
C37A—C38A—C39A—C40A	0.7 (7)	C37B—C38B—C39B—C40B	0.2 (6)
C38A—C39A—C40A—C41A	0.0 (8)	C38B—C39B—C40B—C41B	−0.5 (7)
C39A—C40A—C41A—C42A	−0.6 (7)	C39B—C40B—C41B—C42B	0.5 (7)
C38A—C37A—C42A—C41A	0.1 (6)	C38B—C37B—C42B—C41B	0.0 (6)
N12A—C37A—C42A—C41A	−175.9 (3)	N12B—C37B—C42B—C41B	−178.2 (3)
C40A—C41A—C42A—C37A	0.5 (6)	C40B—C41B—C42B—C37B	−0.3 (6)
